# Unveiling Lived Experiences: Exploring the Health and Lifestyle Effects of COVID-19 on Healthcare Workers

**DOI:** 10.3390/nu15234857

**Published:** 2023-11-21

**Authors:** Rebecca Shenkman, Lisa Diewald, Mary Beth Murray, Tracy L. Oliver

**Affiliations:** 1 MacDonald Center for Nutrition Education and Research, M. Louise Fitzpatrick College of Nursing, Villanova University, Villanova, PA 19085, USA; lisa.diewald@villanova.edu; 2 M. Louise Fitzpatrick College of Nursing, Villanova University, Villanova, PA 19085, USA; mmurra45@villanova.edu (M.B.M.); tracy.oliver@villanova.edu (T.L.O.)

**Keywords:** COVID-19 pandemic, healthcare workers, weight change, physical activity, lifestyle habits, eating patterns

## Abstract

The COVID-19 pandemic brought about significant life disruptions among healthcare workers (HCWs), including changes in weight, eating habits, and physical activity. This qualitative study sought to evaluate the initial and longitudinal effects of health habits among HCWs throughout the pandemic. Data were collected through Qualtrics surveys at three points over a 2-year period with questions asking participants (*n* = 234) to describe whether they experienced changes in weight, eating behaviors, and physical activity and why they believe these changes occurred. The open-ended responses were analyzed following the summative content analysis approach. Four key themes emerged: (1) problematic eating patterns and habits, (2) disruptions in physical activity, (3) alterations in work environment and schedule, and (4) declines in mental health. Respondent reflections highlight the immediate and long-term pandemic-related effects on weight status for some, attributed to alterations in routines and health habits. Other HCWs reported a “reset” or indicated their habits may have been initially disrupted but normalized or improved over the 2-year time span. Findings underscore the need for strategies that support the physical and mental health of healthcare workers.

## 1. Introduction

The coronavirus 2019 (COVID-19) pandemic brought about unprecedented challenges and interruptions in nearly all aspects of life, halting global economies, significantly impacting physical and mental health, changing working patterns, and altering lifestyle habits and routines [1,2,3,4,5,6]. Throughout the pandemic, healthcare workers (HCWs), including nurses and essential workers, experienced unparalleled burdens due to job-related demands of providing continued and essential care to patients while balancing everyday disruptions to daily routines. Life events that induce stress have been observed to affect both physical and emotional well-being through alterations in eating patterns, nutritional choices, physical activity routines, alcohol misuse, and mental and emotional distress [2,4,7,8,9]. The literature on the health impacts on HCWs during the COVID-19 pandemic remains an active field of research as long-term effects remain unknown, but the desire to inform future interventional strategies to enhance the health of HCWs remains high [10,11,12].

Healthy lifestyle behaviors such as maintaining healthy eating habits and participation in physical activity have been associated with higher quality of life among HCWs, which has downstream implications for improved quality of care and professional performance [13,14]. The pandemic, however, introduced numerous stressors and barriers that affected healthy lifestyle behaviors, such as one’s relationship with food and the food environment [15]. Changes in eating patterns can be connected to psychological, social, and emotional health, all of which were undoubtedly impacted during the pandemic [15,16,17]. Furthermore, there is a well-established body of literature highlighting the connection between weight gain and psychological stress arising from work-related and life-related constraints [1,18,19]. Stress and the absence of effective coping mechanisms are often linked to elevated consumption of “comfort foods”, which are generally low in nutritional value but high in sugar and fat content, making them highly palatable [2,20,21].

Prior research has examined the influence of various pandemic-induced factors, such as stress, working conditions, and access to healthy food options, on the eating behaviors and weight management of the adult population [6,22,23,24] and HCWs [25]. Analyzing 23 longitudinal studies conducted in 12 countries, a systematic review offered an all-encompassing insight into the eating behavior traits of adults during the COVID-19 pandemic [22]. The findings indicated a transition towards altered dietary habits, including increased snacking frequency, overindulgence, and a preference for sweets and highly processed foods rather than fresh fruits and vegetables [22]. In addition, a 2023 comprehensive review of 71 studies involving 250,715 participants across 32 countries reported shifts in crucial eating habits in the initial stages of the pandemic [26]. The review suggested that, in general, dietary behaviors were more inclined to stay consistent during the pandemic rather than undergoing significant changes; however, those behaviors that did change predominantly leaned towards increased consumption [26].

The global advent of COVID-19 significantly influenced the lifestyle habits and daily dietary patterns of adults worldwide. While much of the recently published literature captures both quantitative and qualitative data on the impact of the pandemic on dietary habits and weight changes in the general adult population [6,22,23,24,25], few focus exclusively on the HCW population in the United States and none have been found that extend to 2 years. The experiences of this distinct population are exceptional, making it challenging to generalize from existing literature. Therefore, a more comprehensive exploration of healthcare workers’ nuanced experiences at various stages of the pandemic is warranted. This longitudinal qualitative study aimed to properly understand the “why” and “how” for the changes in weight and eating patterns among HCWs that occurred through establishing comparisons over a 2-year period during the pandemic. By identifying core themes driving pandemic-induced changes in weight, eating patterns, and physical activity, the results from this study can inform targeted interventions and policies to mitigate the negative effects of future stressful life events on eating behaviors and weight management among HCWs.

## 2. Materials and Methods

### 2.1. Study Design

The CHAMPS Lifestyle Study (CHAMPS-LS) was a 2-year ancillary study to the longitudinal COVID-19 Study of Healthcare and Support Personnel (CHAMPS; NCT04370821), the parent study [27]. CHAMPS-LS recruited participants from a national registry of HCWs, which included adult (age 18 or older) healthcare personnel, support personnel, and first responders associated with any healthcare facility or as part of the community and directly involved in the care of COVID-19 patients [27]. This paper reports on the qualitative data collected at three time points; baseline (M0—January 2021, first CHAMPS-LS questionnaire deployed, time point one); month 12 (M12—January 2022, time point two); month 24 (M24—January 2023, time point 3) to assess weight change, physical activity, and eating behaviors over time throughout the COVID-19 pandemic. The questionnaires were created using the online survey creation software Qualtrics version 9893. The study was reviewed and approved by the authors’ University Institutional Review Board.

### 2.2. Participants

Participants who participated in the parent CHAMPS Registry during May and June 2020, approximately at the first peak of the pandemic (*n* = 801), were invited by email to participate in the CHAMPS Lifestyle Study deployed on 5 January 2021. Of this pool, 241 consented and received access to the M0 CHAMPS Lifestyle Study questionnaire. Of the 241 who consented, 234 respondents provided complete data at baseline which were used in the analysis; 127 respondents completed the M12 data collection; 80 respondents completed the M24 data collection. No compensation was provided for participation.

The baseline sample of 234 study participants included 203 women (86.8%), 30 (12.8%) men, and 1 (0.4%) gender non-conforming individual. The mean age was 38.69 ± 12.09 years, and the majority (215, 91.9%) identified as white/non-Hispanic, and the majority were registered nurses (151, 64.5%). See Table 1 for additional baseline demographic information as well as the demographics of the sample at M12 and M24.

### 2.3. Measures

Qualitative research methods provide a valuable opportunity to delve into the lived experiences, perceptions, and attitudes of HCWs, offering rich insights into the complex interplay between individual, social, and environmental factors influencing weight and eating patterns during the COVID-19 pandemic. Open-ended questions at all three time points (M0, M12, and M24) asked participants to describe whether and how their weight and lifestyle behaviors (i.e., eating patterns, physical activity) changed due to COVID-19. These questions were included to expand upon information not captured by the structured quantitative questions. For example, these open-ended responses explored the participants’ opinions on why their weight fluctuated, why/whether their portion sizes changed, and how the COVID-19 pandemic affected participants’ eating habits, food purchasing habits, physical activity, weight, or thoughts related to their health.

### 2.4. Analytic Strategy

Open-ended responses were analyzed following the summative content analysis approach [28]. Three members of the research team initially coded data to identify themes from individual responses. The qualitative responses were first read and independently coded by each research team member. These preliminary themes were then evaluated, cross-compared between members, and themes were finalized following discussions to address discrepancies. Primary themes (coded greater than or equal to 10% of all coded responses across all timepoints) and secondary themes (coded less than 10% of all coded responses across all timepoints) were identified for the open-ended questions.

## 3. Results

### 3.1. Open-Ended Questions

Participants were offered the opportunity to respond to three open-ended questions during a two-year period, which were analyzed to identify themes. The first question, “Please describe why you think your weight changed”, received a total of 130 responses (47 at baseline, 44 at M12, and 39 at M24). The second question, “If you would like, please explain how your eating habits have, have not, or partially returned to what they were prior to the COVID-19 pandemic”, was completed by 75 participants, with 38 at M12 and 37 at M24. The last question, “If you would like, please explain how your physical activity level has, has not, or partially returned to what it was prior to the COVID-19 pandemic”, was answered by 70 respondents (42 at M12 and 28 at M24). These responses were analyzed and coded to identify primary and secondary themes.

### 3.2. Primary Themes

**Figure 1:**
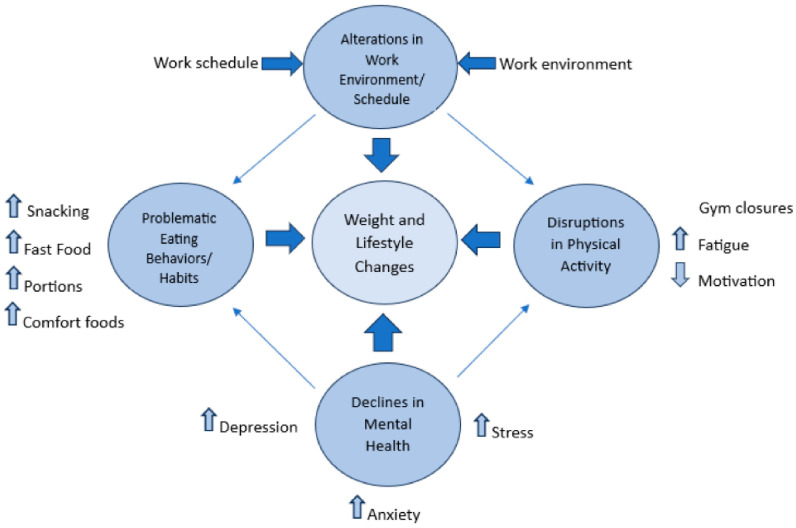
Key themes endorsed by healthcare workers on factors contributing to weight and lifestyle changes during COVID-19.

Our results indicated that, among the respondents, regardless of time point, the pandemic influenced health-related behaviors in a variety of ways. The open-ended questions provided valuable insights into the effects of the disruption on health-related behaviors. Upon analysis of all open-ended questions, four primary themes and four secondary themes emerged, identifying influences of the pandemic on health factors such as eating habits, physical activity, and weight changes. Figure 1 is a pictorial representation of the themes as contributing factors to the weight and lifestyle changes reported by our sample. Additionally, Table 2 represents the primary and secondary themes by timepoint as reasons for unintentional weight change.

#### 3.2.1. Problematic Eating Patterns and Habits

As expected, most participants found that the pandemic resulted in a disruption in normal routines, which in some cases had an impact on weight, eating behaviors, and physical activity. The extent of the impact varied among individuals, and the way in which unhealthy eating patterns and habits manifested themselves varied as well. Some participants reported an increase in snacking. Snacking often took the form of comfort or emotional eating, indulging in salty snacks, high-fat foods, and sweets. Other respondents found themselves snacking more in the evening or enjoying sweets and snacks brought into their workplace by well-meaning community members or patients.

“*I was consistently plant-based prior to COVID. Then ice cream and chips became the only way I could tolerate my grief and fear. I recently regained my control over sugar consumption. I still struggle to reduce consumption of salty crunchy snacks.*”

(M12 participant)

The loss of routine and structure was distressing for some, and accepting this loss and adapting to new meal preparation and eating times was difficult. As a result, as a stop gap measure, some participants noted an increased reliance on fast foods and take out foods rather than cooking at home, and these practices were associated with unwanted weight gain or disruptions in eating habits:

“*There was a lot of free food offered to my unit. Also, I stress eat.*”

(M0 participant)

“*At the beginning of the pandemic, my husband and I were ordering out a lot due to work, exhaustion, wanting to help the community, and we were in the process of moving.*”

(M12 participant)

“*At first I was trying to lose weight, binge eating, lots of take out due to social isolation and being bored, rewarding myself, so I gained 10–15 pounds.*”

(M24 participant)

Some respondents noted that they found themselves eating larger portions than usual, another manifestation of the disruption in usual eating patterns.

“*Prior to the pandemic, I ate pretty healthy meals and portion sizes were appropriate. Al-though not often, now less of an ability to control eating habits, sometimes overeating or eating larger portions.*”

(M12 participant)

The impact of emotional eating and stress-related eating was reported by some respondents, with food becoming a proxy for comfort.

“*I used food for comfort and pleasure because experiencing so little of either due to social isolation of pandemic.*”

(M0 participant)

#### 3.2.2. Disruptions in Physical Activity

Some respondents relied heavily on fitness centers, gyms, and classes for their physical activity at structured times and locations and when these were no longer available, many abandoned physical activity altogether or dramatically reduced it, highlighting again the impact of a disruption in usual routines on health promoting lifestyle habits.

“*I ate more junk and gave up completely on exercising mostly due to overall fatigue, physical and emotional and also because I was showering before and after work and did not want to shower a third time during the day.*”

(M0 participant)

“*The gym I attended decided to require everyone to wear masks while they worked out. I could not wear a mask while doing my HIIT/cardio routine and I was “mask-shamed”… I wanted to put my membership on hold, but the gym just canceled my membership instead.*”

(M12 participant)

“*I was working out less… too mentally and physically exhausted.*”

(M12 participant)

“*I was more active before the pandemic, going to the gym. Now I don’t do anything.*”

(M24 participant)

#### 3.2.3. Declines in Mental Health

There were also stressors that were noted and intertwined with other themes throughout the time points that were related to changes in mental health or sleep routines, which appeared to be a driving force that may have negatively impacted their health behaviors.

“*Constant stress distracted me constantly from my goals. Everyday adaptations to what is going on with COVID has had a huge impact on my ability to focus and perform.*”

(M0 participant)

#### 3.2.4. Alterations in Work Environment or Schedule

It was evident from participants’ responses they endured changes in the work environment, including disruptions in usual shift/hours and work expectations which lessened their ability to prioritize self-care. Motivation for following up on previously established healthy eating and PA goals and routines declined. The constant need to adapt to these changing circumstances was evident in their responses:

“*Prior to COVID I ate a great diet. Mostly homecooked meals, fruit, veggies. In the past 6 months I have been working more hours and unpredictable hours and I just ate what is available to eat which is usually not good stuff and I have less time to cook.*”

(M0 participant)

“*I had difficulty staying motivated due to job stress and exhaustion. We were short staffed, working harder and longer hours.*”

(M12 participant)

### 3.3. Secondary Themes

#### 3.3.1. Improvements in Physical Activity

While most participants reported a negative influence on eating and physical activities, some used the disruption to their advantage, or learned to accept and adapt to change over time. Others found lingering issues with mental health made it exceedingly difficult to resume healthier lifestyle habits. The ability for some to “reset” was noted primarily by M12 and M24 respondents in their comments related to physical activity and having more time at home to be physically active.

“*My activity level has varied. Initially slowed due to a fear of going to the gym and closures. Found alternative ways to exercise, some have sustained.*”

(M12 participant)

“*I have more structured meals and a bit more flexibility to go for walks mid-day.*”

(M12 participant)

#### 3.3.2. Intentional Healthier Lifestyle Habits

For some, there were intentional improvements related to adopting a healthier lifestyle and these often took the form of either small changes in eating habits, such as more meal preparation at home and/or a return to healthier snack choices.

“*We have been more focused on meal prepping and eating healthier at home. Some weeks are better than others.*”

(M12 participant)

“*Since I stopped exercising, I have snacked more, but I try to keep the snacks healthy, such as carrot sticks, nuts, fruit. I noticed weight gain and I increased my water intake. Instead of cutting my tea with water, I just drink plain water. I am making more salads. If I have a calorie heavy meal one day the next day, I try to have a salad.*”

(M12 participant)

“*There were times during the pandemic that we had increased take out. That has decreased to about 1 time per week. Now that our daughter is starting to try foods, we have a lot more fruits and vegetables in the home. That is helping keep us healthier too.*”

(M24 participant)

“*Before I drank a soda daily and would have a bite size piece of candy. But since I have gained weight, I cut out soda and candy.*”

(M24 participant)

#### 3.3.3. Changes in Sleep/Fatigue

Disruptions in normal sleep patterns, especially when combined with other interruptions in work or home routines, resulted in unintentional weight changes for some and/or choosing the more convenient but perhaps less healthy food choices.

“*(I think my weight changed) because of the stress and working too much and not enough sleeping due to working both day and night.*”

(M0 participant)

“*Lack of sleep and lack of time caused me to eat out frequently and eat far less healthy than if I cooked myself.*”

(M12 participant)

#### 3.3.4. Other

Preexisting medical conditions, changes in health (COVID-related or unrelated), as well as pregnancy or breast feeding were mentioned by some participants as factors affecting weight changes, eating habits and/or physical activity.

“*I had a concussion during the first part of the pandemic and after PT I never really resumed regular exercise.*”

(M12 participant)

“*I lost 25 pounds after the first COVID diagnosis. Loss of taste and smell for a few months really curbed appetite and intake. But all the weight returned over the last 20 months and my second bout with COVID did not result in any loss of senses.*”

(M24 participant)

### 3.4. Final Thoughts Related to Changes in Lifestyle Habits

Lastly, an open-ended question asked for any final thoughts, specifically, “Please feel free to share more about how the COVID-19 pandemic affected your eating habits, food purchasing habits, physical activity, weight, or general health in the block below. Your insights are important in helping us learn more about how lifestyle and health habits are impacted during pandemic periods”. This question was intended to allow participants to elaborate on their stories and illustrate the factors they felt contributed to their overall changes in habits. This provided an important context to the story and gave participants a voice to share their personalized experiences and detailed accounts of physical, psychological, and social health habits consequences of the pandemic. Eighteen participants answered this question at both baseline and 24 months. Of particular interest were the nuanced topics mentioned that also deserve attention but that were not identified as key themes, including professional burnout and disordered eating patterns. The prolonged and intense nature of the pandemic contributed to feelings of burnout, prompting responses like “we have all gone from healthcare heroes to punching bags who have to tolerate and overcome daily abuse” and expressed frustration that “health care employees still do very little to take care of their staff”. Others noted the pandemic induced a resurgence of eating disorders and/or disordered eating, saying, “I weigh less now because it’s one of the risks I can control”, and “I have a history of binge eating and bulimia when I was younger, I have been having a problem with this […] again”. All the responses are critical to understanding the larger narrative of how pandemic-induced lifestyle disruptions shape physical and mental health behaviors and outcomes of HCWs, and how best to prevent a repeat situation in the future.

## 4. Discussion

The COVID-19 pandemic has had a profound impact on HCWs, not only in terms of their professional duties but also on their personal health and well-being. This study addressed a unique research gap among HCWs in the United States. By way of a 2-year longitudinal study, this study provided continuous monitoring of the perceived influences of the COVID-19 pandemic on weight changes and lifestyle behaviors, in addition to documenting health-related experiences, challenges, and coping strategies of HCWs. These HCW reflections captured not only upon the initial reactions during the pandemic but how behaviors may—or may not—have rebounded over time.

The lifestyle health consequences of COVID-19 on HCWs have been significant and continue to be a major concern. The pandemic placed extraordinary stress and physical and emotional burden on these frontline workers, leading to a range of health challenges and consequences. No qualitative study to date has extended this long and focused exclusively on pandemic-induced weight change and lifestyle behaviors of HCWs in the United States. To this end, comparisons of data are largely, but not solely, limited to cross-sectional or shorter-term longitudinal studies assessing the eating and nutritional habits of the adult population in general or HCW populations outside the United States. A review of this literature does support similar themes associated with reasons for weight, eating patterns, and physical activity changes, such as stress, changes in physical activity levels, and adoption of unhealthy eating behaviors to alleviate negative emotions after working in a stressful environment [2,6,29,30,31,32,33]. Two internationally based cross-sectional studies that included an examination of eating and nutritional habits among HCWs paralleled our key themes by finding stress and emotional eating were related to changes in dietary habits and a general decline in overall quality of nutritional intake [32,33]. Our respondents also indicated pandemic-related changes like work-related demands and schedules led to a disruption in routine, specifically with meal consistency at work and/or at home. In some cases, this was due to working longer hours, extended intervals during meals, and/or sleep schedule changes with a subsequent loss of usual meal schedules and alterations in meal composition. Meal timing, such as delayed or missed meals and late-night eating, may disrupt circadian rhythms for some individuals, which may lead to an increased risk for obesity or other unhealthy consequences [34,35,36]. While the findings of this study do not suggest causality between meal timing effects and schedule consistency related to weight change, our findings do support research that suggests that the timing of when we eat can have an impact on health and weight, perhaps as much as the quantity and quality of the food we eat.

Our study indicates that working under highly challenging conditions affected the perceived health of HCWs through alterations in eating habits, physical activity, unintended weight loss or gain and effects of stress. It has been observed that HCWs experienced a higher level of psychological distress compared to the general population, resulting in increased rates of depression, stress, anxiety, emotional and physical exhaustion (i.e., burnout), and sleep disturbances [10,37,38,39,40]. Burnout among healthcare workers during the COVID-19 pandemic has been a significant and widespread concern. Healthcare professionals have been at the forefront of the response to the pandemic, facing excessive work, overwhelming personal demands, and/or continuous stress which has led to high levels of burnout [37,38]. It was interesting that the word “burnout” did not explicitly appear in our open-ended responses, yet its essence was woven throughout our responses and echoed in our thematic findings, which reflected some of the typical health outcomes of burnout, such as reduced physical activity, unhealthy eating behaviors, and stress [41,42]. In addition, it is crucial to acknowledge the potential ramifications of the pandemic on individuals with preexisting mental health conditions, including eating disorders. It is evident from emerging research that individuals with eating disorders and/or disordered eating have faced exacerbated symptoms during the COVID-19 pandemic [43,44]. The heightened stress, isolation, and disruptions in daily routines have triggered disordered eating behaviors among this vulnerable population. The pandemic introduced unique triggers, such as fears of weight gain, boredom, loneliness, food shortages, and uncertainty about the future, which may have precipitated the onset or resurgence of eating disorders among some individuals [44].

### 4.1. Next Steps

The COVID-19 pandemic thrusted HCWs onto the frontlines, where they displayed extraordinary dedication and resilience in the face of unprecedented challenges. However, the toll on their physical and mental health has been undeniable. The negative health outcomes and behaviors observed and reported among HCWs, such as weight change, stress, and physical and emotional exhaustion, are alarming indicators of a silent crisis within our healthcare workforce. To preserve the well-being of these essential professionals and ensure the sustainability of our healthcare systems, a multifaceted approach is necessary. The pandemic underscored the importance of recognizing and addressing the physical and mental health needs of HCWs especially, but not limited to, high-stress periods.

While many of the responses received demonstrated a negative impact of the pandemic on healthy lifestyle behaviors, our study did uncover instances in which pandemic-related disruptions and challenges increased resilience, allowing some participants to over time to 0 circumstances and make adaptations to minimize the impact on health and, in some cases, boost health-promoting behaviors. Additional research on factors affecting resiliency in HCWs can potentially lead to interventions that lead to improvements in coping.

Expanding employee wellness programs, or developing interventions focused on developing positive coping strategies in HCWs to facilitate resilience and adaptability are warranted to reduce the impact of stress on overall health and well-being. Peer support, mental health resources, and initiatives to address burnout are examples of solutions to help professionals cope with the demands of their work.

### 4.2. Limitations

There are several limitations worth noting for this study. The first limitation was the relatively homogenous sample that lacked gender, racial, ethnic, and occupation diversity, which may limit the generalizability of the study’s findings. Additionally, this lack of diversity likely underrepresents the experiences of HCWs identifying as ethnic and/or racial minorities during the COVID-19 pandemic. All measures in this study were self-reported and may be subject to self-report bias. Furthermore, attrition occurred across the timepoints from M0, M12, and M24, and thus the nonresponse of initial participants may have led to different findings.

## 5. Conclusions

The health and well-being of our healthcare workforce are inextricably linked to the health and well-being of our communities, and the COVID-19 pandemic cast a spotlight on the intersections of physical, mental, and public health. This qualitative study elucidated the unique challenges faced by HCWs in maintaining a healthy lifestyle and the impact of pandemic-related disruptions on a variety of lifestyle factors influencing health. Addressing these challenges requires a multifaceted approach that encompasses mental health support, workplace changes, education, and policy initiatives. By prioritizing the well-being of HCWs, we can better prepare and support this essential workforce not only during future public health crises, but every day.

## Figures and Tables

**Table 1:** Demographic characteristics of a subsample of HCW participants (*n* = 234).

Variables	Baseline/M0 (*n* = 234)	M12 (*n* = 127)	M24 (*n* = 80)
**Gender**			
Men, *n* (%)	30 (12.8)	15 (12%)	9 (11%)
Women, *n* (*n*%)	203 (86.8)	112 (88%)	71 (89%)
Gender non-conforming individuals, n (%)	1 (0.4)		
Age, mean (SD), years	38.69 (12)	38.94 (12.1)	37.96 (11.9)
**Race**	***n* (%)**	***n* (%)**	***n* (%)**
Black/African American	6 (2.6)	4 (3.1)	3 (3.8)
Latinx/Hispanic	2 (0.9)	0 (0)	0 (0)
White/Non-Hispanic	215 (91.9)	119 (93.7)	73 (91.3)
Asian/Pacific Islander	4 (1.7)	1 (0.8)	1 (1.2)
Multi-racial/Mixed ethnicities	5 (2.1)	2 (1.6)	2 (2.5)
Other	2 (0.9)	1 (0.8)	1 (1.2)
**Body Mass Index**	(*n* = 189) *	(*n* = 120) *	(*n* = 75) *
	***n* (%)**	***n* (%)**	***n* (%)**
Underweight	2 (1.1)	3 (2.5)	1 (1.3)
Normal	80 (42.8)	53 (44.2)	31 (41.3)
Overweight	67 (35.8)	35 (29.2)	21 (28)
Obesity	38 (20.3)	29 (24.2)	22 (29.3)
BMI, mean (SD)	26.64 (6.29)	27.00 (6.98)	27.62 (6.82)
**Job Role**	***n* (%)**	***n* (%)**	***n* (%)**
Nurse (e.g., including RN, CRNP, CRNA, Nursing Assistant, LPN)	151 (64.5)	85 (66.9)	51 (63.75)
Aide or Medical Assistant	5 (2.1)	3 (2.4)	2 (2.5)
Emergency Services (including Police Officers, Paramedics, EMTs, AEMTs, and EMRs)	21 (9.0)	9 (7.1)	7 (8.75)
Therapists (including PT, OT)	9 (3.9)	6 (4.7)	4 (5.0)
Pharmacist/Pharmacy technician	7 (3.0)	7 (5.5)	6 (7.5)
Physician/Physician Assistant	9 (3.8)	6 (4.7)	2 (2.5)
Staff (Reception/Unit Clerk/Administrative Assistant/Housekeeping)	2 (0.8)	1 (0.8)	0 (0)
Registered Dietitian	4 (1.7)	2 (1.6)	2 (2.5)
Social Work	10 (4.3)	5 (3.9)	3 (3.75)
Other (please describe)	16 (6.8)	3 (2.4)	3 (3.75)

* Individuals reporting pregnancy were not included in BMI/weight calculations.

**Table 2:** Number of times primary and secondary themes were coded by time point and by percentage of total coded responses as reasons for weight and lifestyle changes during the COVID-19 pandemic (note: primary themes in bold type with asterisk).

Theme	Description	M0 *n*	M12 *n*	M24 *n*	% of Total Response (*n* = 157)
**Problematic eating behaviors** and habits *	Emotional/stress eating, eating for comfort	18	19	5	26.8%
Disruptions in physical activity *	Gyms closed; schedule disruptions	14	11	6	19.7.%
Declines in mental health *	Increase in stress, depression, anxiety	12	8	2	14%
**Alterations in Work** Environment or Schedule *	Change in work environment/ location	10	6	1	10.8%
Improvements in physical activity	Schedule permitted more physical activity; motivation	5	2	4	7%
Intentional healthier lifestyle habits	Purposeful introduction of healthier eating and lifestyle habits	4	5	1	6.4%
Change in sleep/fatigue	Disruptions in normal sleep habits	3	5	1	5.7%
Other	Medical changes, pregnancy, breastfeeding	4	4	7	9.6%

## Data Availability

The data presented in this study are available on request from the corresponding author. The data are not publicly available due to privacy.
